# Detecting sequence dependent transcriptional pauses from RNA and protein number time series

**DOI:** 10.1186/1471-2105-13-152

**Published:** 2012-06-28

**Authors:** Frank Emmert-Streib, Antti Häkkinen, Andre S Ribeiro

**Affiliations:** 1 Computational Biology and Machine Learning Lab, Center for Cancer Research and Cell Biology, School of Medicine, Dentistry and Biomedical Sciences, Queen’s University Belfast, Belfast, UK; 2 Laboratory of Biosystem Dynamics, Computational Systems Biology Research Group, Department of Signal Processing, Tampere University of Technology, Tampere, Finland

## Abstract

**Background:**

Evidence suggests that in prokaryotes sequence-dependent transcriptional pauses affect the dynamics of transcription and translation, as well as of small genetic circuits. So far, a few pause-prone sequences have been identified from in vitro measurements of transcription elongation kinetics.

**Results:**

Using a stochastic model of gene expression at the nucleotide and codon levels with realistic parameter values, we investigate three different but related questions and present statistical methods for their analysis. First, we show that information from in vivo RNA and protein temporal numbers is sufficient to discriminate between models with and without a pause site in their coding sequence. Second, we demonstrate that it is possible to separate a large variety of models from each other with pauses of various durations and locations in the template by means of a hierarchical clustering and a *random forest* classifier. Third, we introduce an *approximate likelihood function* that allows to estimate the location of a pause site.

**Conclusions:**

This method can aid in detecting unknown pause-prone sequences from temporal measurements of RNA and protein numbers at a genome-wide scale and thus elucidate possible roles that these sequences play in the dynamics of genetic networks and phenotype.

## Background

Noise is inherent in gene expression and affects the behavior of genetic circuits and thus phenotype determination. It is unknown to what extent this noise is evolvable. One mechanism that likely contributes to transcriptional noise in prokaryotes is RNA polymerase (RNAP) pausing during elongation [1,2]. Pausing enhances the propensity for collisions between consecutive RNAPs in the template [3] and, in some cases, of premature terminations [4], particularly when hairpin loops form in the transcript, facilitating the recruitment of Rho-factor, a protein that dissociates the RNA from the DNA template and RNA polymerase [5]. The distance of the hairpin from the RNA 3^*′*^ end and the RNA sequence affect pause duration and proneness for premature termination [4], indicating that the kinetics of this process is sequence dependent [6]. This feature makes transcriptional pausing a plausible mechanism via which natural selection may act upon noise in gene expression.

Long-duration pauses usually occur only at specific DNA sequences [4], while short-duration pauses occur at random locations [7]. Observations in bacteria suggest that the RNAP pauses, on average, every 100 to 200 bp, for a few seconds [6] and, less frequently, for one to several minutes [4].

One of the best studied long-pause sites is the *his* pause sequence. This sequence causes the RNAP to pause for, on average, 47 s, with an efficiency that can go up to 80% [5]. The long duration of this event relies on the formation of a hairpin loop in the elongating RNA sequence that stabilizes the RNAP [7]. By removing the region of the DNA that codes for loop, the duration is reduced to 4.6 s, and becomes exponentially distributed [7]. Unlike *his* pauses, other sequence dependent long pause sites do not require the formation of RNA secondary structures [8].

Studies of transcriptional pausing have focused on the physical-chemical causes and its physiological role in gene expression [4]. Biochemical experiments to single-molecule measurements suggest that there are several kinds of transcriptional pauses, which differ in the causal mechanism and in duration once occurring [6,9-12]. One hypothesis regarding the role of pausing is that it facilitates the coupling of transcription and translation by halting the RNAP, allowing a translating ribosome to catch up [4]. Recently, it was suggested that pauses affect transcriptional noise [1]. Also, the location of the pause-prone sequence, the duration, and the proneness for pausing influence the extent to which the pause affects the kinetics of RNA production [2]. These effects on RNA numbers may be of relevance in prokaryotes, particularly because RNAs usually exist in very small amounts (from one to a few molecules) [13,14].

So far there are only hypotheses regarding what may be the roles of sequence-dependent pauses on the dynamics of gene expression and genetic circuits [1,4,7]. To determine the role of pauses, a better knowledge is required regarding which sequences enhance the occurrence of pauses. Also, more knowledge is needed on the kinetics of the various pausing mechanisms and on their location in the genome to determine which genes’ expression is affected by pauses. For that, methods are needed to recognize the existence of pauses from temporal gene expression profiles. It is also necessary to identify the sequences responsible for the occurrence of transcriptional pauses.

With this aim, here we investigate whether, from temporal RNA and protein numbers, we can determine if there is a long-duration pause site in the elongation region of a gene. Additionally, we aim to estimate, at least by comparison, the mean duration of a pause and its location relative to the transcription start site. For that, we simulate stochastic gene expression dynamics at the nucleotide and codon levels [3,15-17] of genes whose sequence includes long-duration pause sites that promote the occurrence of pauses with identical kinetics to that of the *his* pause [5]. Namely, we produce temporal series of RNA and protein numbers assuming that these molecules can be detected at the single-molecule level, as soon as they are produced, which is possible using MS2-GFP tagging [18,19] for RNA, and tsr-venus sequences for proteins [20]. We use this information to search for pause sites and characterize their kinetic properties making use of statistical methods for classification using features extracted from time series of RNA and protein numbers.

## Methods

### Modeling gene expression

We use a delayed stochastic model of prokaryotic transcription and translation at the nucleotide and codon level that includes the closed and the open complex formation, stepwise elongation, as well as alternative pathways to elongation, namely pausing, arrests, editing, pyrophosphorolysis, RNA polymerase traffic, and premature termination. Stepwise translation can begin after the formation of the ribosome binding site and accounts for variable codon translation rates, ribosome traffic, back-translocation, drop-off, and trans-translation [3,15].

The dynamics follows the delayed Stochastic Simulation Algorithm [21], which is based on the SSA [22]. The delayed SSA allows an arbitrarily distributed time delay to be associated with the release of each of the reaction products, and consequently it can be used to model non-instantaneous sequences of events, which are expected not to follow the exponential statistics of SSA. We make use of such delays to model, for example, events during the stepwise process of transcription initiation. Each chemical species is a variable of integer value. Time advances at discrete steps and, at each step, a reaction occurs and the number of molecules of the species involved are updated according to the reaction formula. In a delayed event, one or more products are kept on a waiting list until sufficient time has elapsed, after which they are released in the system. Delayed events are represented as *A*→*B* + *C*(*τ*). When this reaction occurs at moment *t*, *B* is instantaneously produced at *t* and *C* is placed on a waiting list until it is released at *t* + *τ*[16,17]. The value of *τ*can be drawn from a specified distribution, each time the reaction occurs. This is the case, for example, for the duration of the open complex formation (reaction 1 in Table 1).

**Table 1:** Reactions and kinetic parameters for the gene expression model

**#**	**Chemical reaction(s)**	**Parameters**
1	$\text{Pro}+\text{Rp}\overset{k_{\mathrm{tc}}}{\to }\text{Rp}\cdot \text{Pro}(\tau_{\mathrm{oc}})$	*k*_*tc*_=0.0245, *τ*_*oc*_∼*N*(40,4^2^)
2	$\text{Rp}\cdot \text{Pro}+{\text{U}}_{\left[1,(\Delta_{\text{P}}+1)\right]}\overset{k_{m}}{\to }{\text{O}}_{1}+\text{Pro}$	*k*_*m*_=150
3	${\text{O}}_{n}\overset{k_{a}}{\to }{\text{A}}_{n}$	*k*_*a*_=150 for *n*>10, *k*_*a*_=30 for *n*≤10
4	${\text{A}}_{n}+{\text{U}}_{n+\Delta_{\text{P}}+1}\overset{k_{m}}{\to }{\text{O}}_{n+1}+{\text{U}}_{n-\Delta_{\text{P}}}+{\text{U}}_{n-\Delta_{\text{P}}}^{R}$	*k*_*m*_=150
5	${\text{O}}_{n}\overset{k_{p}}{\underset{1/\tau_{p}}{⇌}}{\text{O}}_{n_{p}}$	*k*_*p*_=0.55,*τ*_*p*_=3
6	${\text{O}}_{n_{p}}+{\text{A}}_{n-2\Delta_{\text{P}}-1}\overset{0.8k_{m}}{\to }{\text{O}}_{n}+{\text{A}}_{n-2\Delta_{\text{P}}-1}$	*k*_*m*_=150
7	${\text{O}}_{n}+{\text{A}}_{n-2\Delta_{\text{P}}-1}\overset{0.2k_{m}}{\to }{\text{O}}_{n_{p}}+{\text{A}}_{n-2\Delta_{\text{P}}-1}$	*k*_*m*_=150
8	${\text{O}}_{n}\overset{k_{\mathrm{ar}}}{\underset{1/\tau_{\mathrm{ar}}}{⇌}}{\text{O}}_{n_{\mathrm{ar}}}$	$k_{\mathrm{ar}}=2.78\cdot 10^{-4}$, *τ*_*ar*_=100
9	${\text{O}}_{n}\overset{k_{\mathrm{ed}}}{\underset{1/d_{\mathrm{ed}}}{⇌}}{\text{O}}_{n_{\mathrm{corr}}}$	*k*_*ed*_=0.009, *d*_*ed*_=5
10	${\text{O}}_{n}\overset{k_{\mathrm{pre}}}{\to }\text{Rp}+{\text{U}}_{\left[(n-\Delta_{\text{P}}),(n+\Delta_{\text{P}})\right]}$	*k*_*pre*_=1.9·10^−4^
11	${\text{O}}_{n}+{\text{U}}_{n-\Delta_{\text{P}}-1}+{\text{U}}_{n-\Delta_{\text{P}}-1}^{R}\overset{k_{\mathrm{pyr}}}{\to }{\text{O}}_{n-1}+{\text{U}}_{n+\Delta_{\text{P}}-1}$	*k*_*pyr*_=0.75
12	${\text{A}}_{\mathrm{last}}\overset{k_{f}}{\to }\text{Rp}+{\text{U}}_{\left[\text{last},\text{last}-\Delta_{\text{P}}\right]}+\text{mRNA}$	*k*_*f*_=2
13	$\text{mRNA}\overset{k_{\mathrm{dr}}}{\to }\emptyset$	*k*_*dr*_=0.025
14	$\text{Rib}+{\text{U}}_{\left[1,\Delta_{\text{R}}+1\right]}^{\text{R}}\overset{k_{\mathrm{tl}}}{\to }{\text{O}}_{1}^{\text{R}}+{\text{Rib}}^{\text{R}}$	*k*_*tl*_=0.53
15	${\text{O}}_{n}^{\text{R}}\overset{k_{\mathrm{tr}\{A,B,C\}}}{\to }{\text{A}}_{n}^{\text{R}}$	*k*_*trA*_=35, *k*_*trB*_=8, *k*_*trC*_=4.5
16	${\text{A}}_{n-3}^{\text{R}}+{\text{U}}_{\left[n+\Delta_{\text{R}}-3,n+\Delta_{\text{R}}-1\right]}^{\text{R}}\overset{k_{\mathrm{tm}}}{\to }{\text{O}}_{n-2}^{\text{R}}$	*k*_*tm*_=10,000
17	${\text{O}}_{n-2}^{\text{R}}\overset{k_{\mathrm{tm}}}{\to }{\text{O}}_{n-1}^{\text{R}}$	see above
18	${\text{O}}_{n-1}^{\text{R}}\overset{k_{\mathrm{tm}}}{\to }{\text{O}}_{n}^{\text{R}}+{\text{U}}_{\left[n-\Delta_{\text{R}}-2,n-\Delta_{\text{R}}\right]}^{\text{R}}$	see above
19	${\text{O}}_{n}^{\text{R}}+{\text{U}}_{\left[n-\Delta_{\text{R}}-2,n-\Delta_{\text{R}}\right]}^{\text{R}}\overset{k_{\mathrm{bt}}}{\to }{\text{A}}_{n-3}^{\text{R}}+{\text{U}}_{\left[n+\Delta_{\text{R}}-3,n+\Delta_{\text{R}}-1\right]}^{\text{R}}$	*k*_*bt*_=1.5
20	${\text{O}}_{n}^{\text{R}}\overset{k_{\mathrm{drop}}}{\to }\text{Rib}+{\text{U}}_{\left[n-\Delta_{\text{R}},n+\Delta_{\text{R}}\right]}^{\text{R}}$	*k*_*drop*_=1.14·10^−4^
21	$\text{mRNA}\overset{k_{\mathrm{tt}}}{\to }[{\text{Rib}}^{\text{R}}]\times \text{Rib}$	*k*_*tt*_ is sequence dependent
22	${\text{A}}_{\mathrm{last}}^{\text{R}}\overset{k_{tl_{f}}}{\to }\text{Rib}+{\text{U}}_{\left[\mathrm{last},\mathrm{last}-\Delta_{\text{R}}\right]}^{\text{R}}+\text{P}(\tau_{\mathrm{fold}})$	$k_{tl_{f}}=2$, *τ*_*fold*_∼*N*(420,100^2^)
23	$\text{P}\overset{k_{\mathrm{dp}}}{\to }\emptyset$	*k*_*dp*_=0.0029

Chemical reactions, rate constants (in s^−1^), and delays (in s) used to model transcription and translation. Pro – promoter, Rp – RNA polymerase, Rib – ribosome, [Rib^R^ – number of translating ribosomes on RNA strand, P – complete protein, U – unoccupied nucleotide and O – nucleotide occupied by Rp, A – activated nucleotide; U^*R*^,O^*R*^,A^*R*^ – corresponding ribonucleotides. *n* denotes the number of the nucleotide in the sequence. *Δ*_P_ – range of nucleotides that Rp occupies, *Δ*_P_=25. *Δ*_R_ – range of ribonucleotides that ribosome occupies, *Δ*_R_=31. Notation *X*∼*N*(*μσ*^2^) denotes that the values of *X* are drawn from normal distribution with a mean of *μ*and variance of *σ*^2^. Parameter values are from measurements in *E. coli*, mainly for LacZ [3,20,24-34,53]. The duration of protein folding after translation is completed (*τ*_*fold*_) is set according to measurements of a commonly used GFP mutant [35].

The model of transcription accounts for the binding of the RNAP to the template and diffusion along the template (reaction 1 in Table 1), promoter open complex formation (*τ*_*oc*_ in reaction 1) [23], promoter clearance (reaction 2), nucleotide activation followed by stepwise elongation at each nucleotide (reactions 3 and 4, respectively), and termination (reaction 12). The reactions competing with stepwise elongation are transcriptional pauses (reactions 5), collisions between RNAPs, which may release (reaction 6) or induce pauses (reaction 7), arrests (reactions 8), misincorporation and editing (reactions 9), premature terminations (reaction 10), and pyrophosphorolysis (reaction 11). The number of nucleotides (2*Δ*_P_ + 1) occupied by the RNAP on the strand while elongating is 25 [24]. Finally, mRNA can undergo degradation (reaction 13) [15].

The model of translation includes translation initiation (reaction 14 in Table 1) and ribonucleotide activation (reaction 15) followed by stepwise translocation (reactions 16 to 18) [25,36]. Reactions competing with translocation are the back-translocation (reaction 19), ribosome drop-off (reaction 20), and trans-translation (reaction 21). After elongation completion, it follows protein folding (reaction 22). The model accounts for codon-specific translation rates [37] and for the ribonucleotides occupied by a ribosome when on the RNA strand [15]. Finally, protein molecules undergo degradation (reaction 23). Note that each time we generate the sequence of a model gene, we generate the codon sequence randomly, according to the statistical frequency of each codon in *Escherichia coli* (extracted from NCBI GenBank as of Dec. 1st, 2011) [38].

### Modeling sequence-dependent pauses

Two types of transcriptional pauses have been identified: i) ubiquitous pauses, which can occur at any nucleotide with approximately uniform probability of occurrence [7], and ii) sequence-dependent pauses, which occur at specific regions of the sequence [4].

Reaction 5 (forward direction) in Table 1 models the occurrence of ubiquitous pauses and their release (backward direction). To introduce a sequence-dependent pause in nucleotide *n*, we modify the reactions at that location as follows:

(1) $${\text{O}}_{n}\overset{k_{n_{p}}}{\underset{1/\tau_{n_{p}}}{⇌}}{\text{O}}_{n_{p}}$$

where $k_{n_{p}}=k_{a}{\left({\varepsilon_{n_{p}}}^{-1}-1\right)}^{-1}$ is the rate of pausing, $\tau_{n_{p}}$ is the mean duration of the pause, and $\varepsilon_{n_{p}}$ denotes the pause efficiency, that is, the probability that an RNAP pauses when at the *n*th nucleotide.

As specified in reaction 1, the duration of these pauses is randomly drawn from an exponential distribution with the appropriate mean pause duration each time it occurs. It is noted that the assumption of exponential duration of each pause event is based on measurements where the sequence causing the pause is present, but the subsequent sequence where hairpin loops form (stabilizing the paused state) is not [7]. Unfortunately, there are yet no measurements available informing of the distribution of the durations of these long pauses, and thus we opted to make this assumption.

When a pause occurs, the ribosomes translating the RNA proceed only until the point where the RNAP is stranded. At that point, ribosomes pause until the RNAP is released [4]. Due to this, the pauses are expected to affect protein number dynamics [1].

### Detecting the presence of a sequence-dependent pause site

Simulations of the models are initialized without RNA or proteins in the system. For our analyses we use only the stationary part of a time series. The methods assume the time series to be weakly stationary, meaning that the first two moments (i.e. mean and variance) do not vary over time. This condition is, in all cases, tested by a two-sample t-test for the ensemble mean values for a sample size of 10.

We first present a method to detect a sequence-dependent pause site from the time series of RNA and protein numbers. We denote by *R*_*M*_, $R_{M^{\prime }}$ two matrices of size *F*×*L*containing the number of mRNAs generated from two models, *M* and *M*^*′*^. *F* is the number of time series generated for each model and *L* is the length of the part of a time series that is assumed to be stationary. *R*_*M*_(*i*,) is a vector of length *L* containing the number of mRNAs of the *i*th time series for model *M*. Analogously, *P*_*M*_, $P_{M^{\prime }}$ are two matrices of size *F*×*L*containing the number of proteins for the two models. A thorough discussion of the generated data, i.e., how it was sampled, can be found in the results section.

Previous work based on the simulations of stochastic models similar to the one used here [2], reported that the presence of sequence dependent pauses affect the RNA production sufficiently to have a discernible effect on the mean number of mRNAs. We use this feature as a *statistic* to discriminate between models with and without a pause site. More precisely, we conduct hypotheses tests according to the following procedure. First, we estimate the mean number of mRNAs for two models *M* and *M*^*′*^from a randomly sampled time series *i* of length *ΔL*by: 

1. sample *i*∼unif(1:*F*)

2. sample *L*_*s*_∼unif(1:*L*−*ΔL*)

3. estimate the mean number of mRNAs for model M and M’:

(2) $$m_{M}^{R}(s)=\frac{1}{\mathrm{\Delta L}+1}\overset{L_{s}+\mathrm{\Delta L}}{\underset{t=L_{s}}{\sum }}R_{M}(i,t)$$

(3) $$m_{M^{\prime }}^{R}(s)=\frac{1}{\mathrm{\Delta L}+1}\overset{L_{s}+\mathrm{\Delta L}}{\underset{t=L_{s}}{\sum }}R_{M^{\prime }}(i,t)$$

Here, the symbol unif(*x*:*y*) indicates the uniform probability distribution with a discrete domain from *x* to *y*. We repeat the above procedure for *s*∈*S* samples to obtain two profile vectors of dimension *S* containing information about the mean number of mRNAs. Based on the profiles $m_{M}^{R}$ and $m_{M^{\prime }}^{R}$ we conduct a two-sample t-test [39] for their mean values:

(4) $$\begin{array}{ll} \text{Null hypothesis:}\;H_{0}:\mu_{m_{M}^{R}}=\mu_{m_{M^{\prime }}^{R}} & \; \end{array}$$

(5) $$\begin{array}{ll} \text{Alternative hypothesis:}\;H_{1}:\mu_{m_{M}^{R}}\neq \mu_{m_{M^{\prime }}^{R}} & \; \end{array}$$

This test results in a p-value, *p*, indicating for *p*≤*α*the statistical significance of the test, i.e., the rejection of the null hypothesis, for a given significance level *α*. This p-value should be denoted as $p_{M,M^{\prime }}$ since it results from a comparison of data from model *M* and model *M*^*′*^. Repeating the above procedure *N* times results in *N* different p-values that reflect the behavior of the population. Finally, we apply the same procedure to *P*_*M*_and $P_{M^{\prime }}$ to obtain similar information for the protein levels.

### Definition of feature vectors

For each of the models with pauses with distinct kinetic characteristics, we measure the number of mRNAs and of proteins, and the cumulative number of proteins as a function of time, represented by matrices, *R*_*M*_, *P*_*M*_ and *E*_*M*_, respectively. Following the previous notation, each matrix has size *F*×*L*, where *F* is the number of repeated time series and *L* is the length of the stationary time series.

To perform a clustering and a classification of the time series data generated from the different models, we define the following 10 features, which we use to define feature vectors. These features capture information about the autocorrelation, cross-correlation and the duration of the transcription and translation processes. Specifically, we estimate the lag-*l* sample autocorrelation, *r*_*xx*_(*l*) [40,41] by

(6) $$r_{\mathrm{xx}}(l)=\frac{\overset{T}{\underset{t=l+1}{\sum }}(x_{t}-{\bar{m}}_{x})(x_{t-l}-{\bar{m}}_{x})}{\overset{T}{\underset{t=1}{\sum }}{\left(x_{t}-{\bar{m}}_{x}\right)}^{2}}.$$

Here 0≤*l*<*T*−1 and ${\bar{m}}_{x}=\overset{T}{\underset{t=1}{\sum }}x_{t}/T$ is the mean of the time series {*x*_*t*_}. We estimate the lag-*l* sample autocorrelation for *R*_*M*_and *P*_*M*_, i.e., *r*_*xx*_(*l*;*R*_*M*_) and *r*_*xx*_(*l*;*P*_*M*_). Then we estimate the mean and the standard deviation of the autocorrelation function, *r*_*xx*_, up to lag *K* by

(7) $$\begin{array}{ll} m(r_{\mathrm{xx}})=\frac{1}{K}\overset{K}{\underset{l=1}{\sum }}r_{\mathrm{xx}}(l)\times l & \; \end{array}$$

(8) $$\begin{array}{ll} s(r_{\mathrm{xx}})=\frac{1}{K-1}\overset{K}{\underset{l=1}{\sum }}\left(\right)r_{\mathrm{xx}}(l)\times l-m(r_{\mathrm{xx}}){))}^{2} & \; \end{array}$$

For our numerical analysis we set *K*=300. Similarly, we estimate the lag-*l* cross-correlation, *r*_*xy*_(*l*), by

(9) $$r_{\mathrm{xy}}(l)=\frac{\overset{T}{\underset{t=l+1}{\sum }}(x_{t}-{\bar{m}}_{x})(y_{t-l}-{\bar{m}}_{y})}{\sqrt{\overset{T}{\underset{t=1}{\sum }}{\left(x_{t}-{\bar{m}}_{x}\right)}^{2}\overset{T}{\underset{t=1}{\sum }}{\left(y_{t}-{\bar{m}}_{y}\right)}^{2}}},$$

with 0≤*l*<*T*−1 and ${\bar{m}}_{y}=\overset{T}{\underset{t=1}{\sum }}y_{t}/T$ is the mean of the time series {*y*_*t*_}. Also, for the cross-correlation function we estimate *m*(*r*_*xy*_) and *s*(*r*_*xy*_) up to lag *K* for *R*_*M*_and *P*_*M*_.

Further, we estimate the mean decay time of the transcripts and its standard deviation. To obtain these, we first determine a vector, *d*, of decay times of mRNAs by estimating for how many consecutive steps

(10) $$R_{M}(i,t-1)\geq R_{M}(i,t)$$

holds during the time series *R*_*M*_(*i*,). A component of vector *d* therefore gives the number of consecutive steps for which the number of mRNAs does not increase. From the resulting vector *d*(*R*_*M*_(*i*,)) we estimate its mean, *m*(*d*(*R*_*M*_(*i*,))), and standard deviation, *s*(*d*(*R*_*M*_(*i*,))).

A summary of all 10 variables is given in Table 2. We use these variables to define a 10 dimensional feature vector $v_{M}\in R^{10}$ for a model *M*, i.e., *v*_*M*_(*i*) gives the value of the *i*-th variable in Table 2.

**Table 2:** Features used for classification

**#**	**Feature**	**Description**	**Data**
1	*m*(*r*_*xx*_;*R*_*M*_)	mean autocorrelation function	*R*_*M*_
2	*s*(*r*_*xx*_;*R*_*M*_)	standard deviation of autocorrelation function	*R*_*M*_
3	*m*(*r*_*xx*_;*P*_*M*_)	mean autocorrelation function	*P*_*M*_
4	*s*(*r*_*xx*_;*P*_*M*_)	standard deviation of autocorrelation function	*P*_*M*_
5	*m*(*r*_*xy*_;*R*_*M*_,*P*_*M*_)	mean cross-correlation function	*R*_*M*_ and *P*_*M*_
6	*s*(*r*_*xy*_;*R*_*M*_,*P*_*M*_)	standard deviation of cross-correlation function	*R*_*M*_ and *P*_*M*_
7	*m*(*r*_*xy*_;*P*_*M*_,*E*_*M*_)	mean cross-correlation function	*P*_*M*_ and *E*_*M*_
8	*s*(*r*_*xy*_;*P*_*M*_,*E*_*M*_)	standard deviation of cross-correlation function	*P*_*M*_ and *E*_*M*_
9	*m*(*d*(*R*_*M*_))	mean decay time	*R*_*M*_
10	*s*(*d*(*R*_*M*_))	standard deviation of decay time	*R*_*M*_

Summary of the 10 variables we use to define a feature vector for a model *M*.

We would like to emphasize that all three types of measures introduced above, based on autocorrelation, cross-correlation and the decay time, are fundamentally different from each other. Whereas the first two types of measures are based on a different usage of correlation coefficients within (nr. 1, 2, 3, 4 see Table 2) and between time series (nr. 5, 6, 7, 8 see Table 2), the latter measure is not referential. Instead, it provides information about the continuity of the transcription process. In the results section, we will provide quantitative information for this argument.

## Results and discussion

We model genes 1,000 nucleotides long. Unless otherwise stated, the long-pause site is at nucleotide 500 and has the same kinetic properties as a *his* pause, i.e., the efficiency of pausing is $\varepsilon_{n_{p}}=0.8$ (measurements indicate that it ranges from 0.5 to 0.8 [5]) and the mean duration is $\tau_{n_{p}}=47$ s [5]. We do not model an enhancement in the premature terminations at this location, since measurements of the kinetic properties of this process are not yet available. However, the occurrence of pauses may nevertheless lead to an increase of premature terminations due to increasing the expected duration of the elongation process which, on its own, may lead to an enhanced chance of premature termination of RNAPs preceding the paused one [1]. The models are implemented and simulated in SGN Sim [17].

For the following analysis, we consider six models, A through F, described in Table 3. In the null model A, we assume ubiquitous pauses only. Namely, at each nucleotide there is a rate of occurrence of pausing set to 0.55 s^−1^. Once occurring, such pauses last, on average, 3 s following an exponential distribution [7].

**Table 3:** Six different models used for detection of pauses

Model	Features
A	No sequence-dependent pause sites.
B	Pause site at nucleotide 500.
C	Pause site at nucleotide 250.
D	Pause site at nucleotide 750.
E	Pause site with mean duration $\tau_{n_{p}}=23.5$ s at nucleotide 500.
F	Pause site with mean duration $\tau_{n_{p}}=94$ s at nucleotide 500.

The six models with different pause characteristics are considered for the purposes of detection and classification of sequence dependent pauses.

The comparison between models A and B tests if the presence of a long pause is detectable from time series of RNA and protein numbers. The other models are used to test whether the location and kinetic properties of the pause can be classified. For each model, we simulate 10 instances, each for 1,000,000 s. The sampling frequency of the number of RNAs and proteins is 1 s^−1^. The different instances of each model differ in the codon sequences, as these are randomly generated as described in the Methods section. However, it is noted that the length of the sequence used here was found to be sufficient to not expect significant differences in the kinetics of translation elongation due to differences in the codon sequence.

We found that for *t*≥50,000 s the time series for the models A through F are weakly stationary, as hypothesized by the methods. Additionally, the time series appear *ergodic*, i.e., the ensemble average over different realizations corresponds to the time average over an individual time series [42]. Despite these properties, the average number of proteins estimated from a (small) sample size *S*≪1,000,000 is not a reliable variable that could serve as a feature, e.g., for clustering or classification of different models.

**Figure 1:**
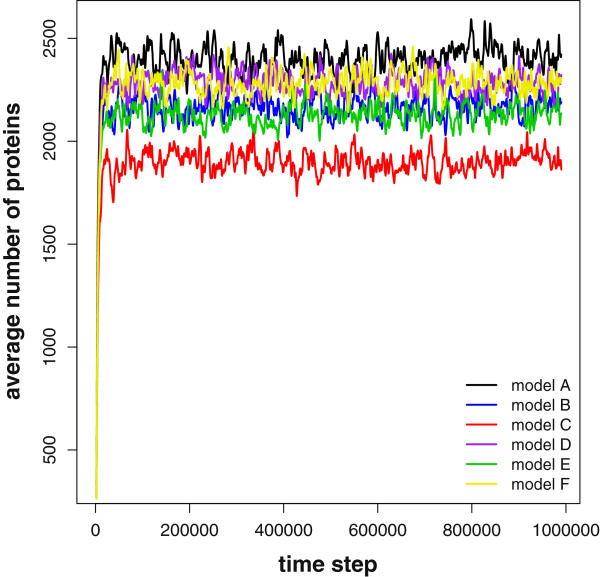
**Average number of proteins.** Average number of proteins for each model. Each time series has been averaged over 10 independent runs and each data point has been averaged over 100 time steps and smoothed over a window of size 20.

To visualize this problem, Figure 1 shows time series of the average of the number of proteins of 10 independent simulations for each model. In addition, each data point has been averaged over 100 time steps and smoothed over a window of size 20. For the smoothing, we used a standard cubic spline smoothing [43]. Despite the smoothing, the resulting time series fluctuate clearly around the mean value of the time series, showing that the average number of proteins from ‘small’ samples is not a reliable feature.

We would like to emphasize that, theoretically, different models can be distinguished from each other by calculating the *asymptotic* average number of proteins, however, in practice (i.e., for small sample sizes *S*≪1,000,000) the fluctuations increase the uncertainty of these estimates. This is especially important if one aims at studying the dynamics of expression of real genes since, given the present methods, asymptotic results are unreachable.

### Detecting a sequence-dependent pause site

First, we test if a sequence-dependent pause with the aforementioned characteristics is detectable. Such a detection would discriminate a model with a pause site from a model without one. To study this, we compare model A with model B with the hypotheses tests described in the methods section.

**Figure 2:**
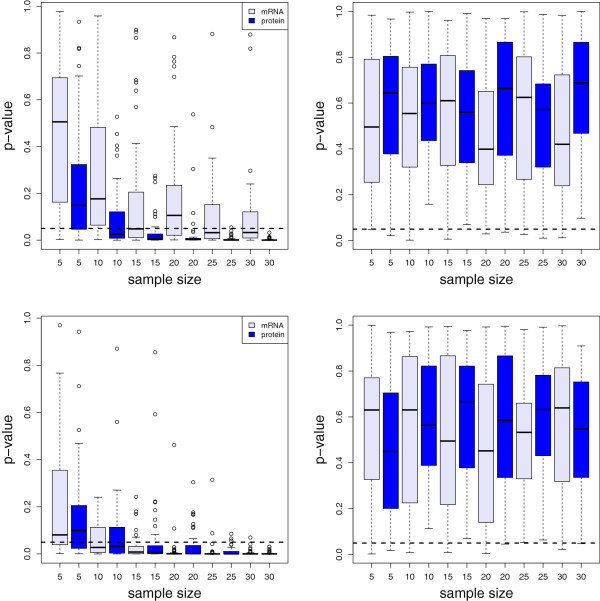
**P-values for comparing model A and B.** P-values in dependence of the sample size from two-sample t-tests. Top row: *ΔL*=200. Bottom row: *ΔL*=1,000. First column: comparison of model A with model B. Second column: comparison of different instances of model A.

The results of the analysis are shown in the first column in Figure 2. There, the distribution of p-values is shown in dependence on the sample size *S* (x-axis) and the length of the time series (*ΔL*). The top row shows results for *ΔL*=200 and the bottom row for *ΔL*=1,000, for illustration purposes. The results correspond to *N*=50, which means that for each sample size, we obtained 50 independent p-values. In general, in a boxplot a ’circle’ corresponds to an outlier.

It is visible that, with larger sample sizes, the median p-values fall below the *α*=0.05 significance level (horizontally dashed line), which means that the two models can be discriminated from each other in a statistical manner. The p-values for *ΔL*=200 are in general higher than for *ΔL*=1,000, as expected, because a shorter time series contains more variability with respect to the estimation of the mean number of mRNAs or proteins.

It is interesting to note that the information provided by the protein level allows a better discrimination for *ΔL*=200 compared to the mRNA level. Specifically, for sample size 10, the median p-value of the number of proteins is clearly significant, whereas the p-value for the mRNAs is not. For longer time series this difference vanishes. Further increasing *ΔL* leads to an even better distinction between the model A and B by requiring a smaller number of samples (not shown).

To demonstrate that the null hypothesis is not rejected if the data come from the same model, i.e., when the null hypothesis is true, we repeat the above analysis to obtain p-values for the cases *p*_*A*,*A*_ and *p*_*B*,*B*_. The second column in Figure 2 shows the result for a comparison of data from model A. In this case, the probability to reject the null hypothesis falsely is very low, because almost all p-values are much larger than our significance level of *α*=0.05. For model B we obtain qualitatively similar results (not shown).

### Classification of models

We hypothesize that despite the intricate dynamics of the gene expression model where, e.g., RNAPs can bump into each other causing mutual delays of transcription, the information captured on the mRNA and protein numbers suffices to distinguish models with different parameter configurations. To demonstrate this, we estimate feature vectors for each model, based on the 10 features defined in the methods section, and show numerically their discriminative power.

The rationale of the following analysis is, first, to use an unsupervised clustering analysis to demonstrate that our features are not only sufficient to recover different models in an unsupervised manner but also that such clusters are robust. Second, we use a random forest classifier to classify the models based on our feature vectors. This allows a precise quantification of the errors made by such a categorization.

**Figure 3:**
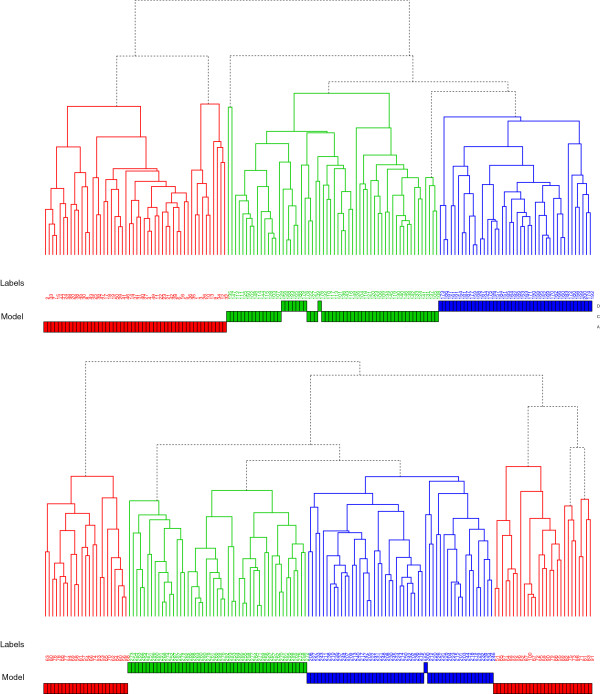
**Results of hierarchical clustering of the models.** Hierarchical clustering of feature vectors from models B, E and F (left tree) and from models A, C and D (right tree). The labels index the feature vectors. Each model is represented by 50 feature vectors.

First, we perform an unsupervised clustering analysis. Specifically, we generate for models A through F time series data from which we estimate 50 feature vectors ${\left\{v_{M}[j]\right\}}_{j=1}^{50}$ for each model. Each of these feature vectors *v*_*M*_*j* is 10 dimensional, i.e., *v*_*M*_*j*(*i*) with *i*∈{1,…,10}. Since the 10 variables defining the components of the feature vectors *v*_*M*_*j*(*i*) are on different scales, we perform a z-transformation separately for each component to scale the different variables. That means, after the z-transformation, every variable (component of a feature vector) follows a standard normal distribution, i.e., *v*_*M*_*j*(*i*)∼*N*(*μ*=0,*σ*^2^=1). Here, the symbol ’∼’ indicates that the random variable (left side) is sampled from a model (right side). To these feature vectors (profiles), we apply a hierarchical clustering using a Manhattan distance measure and the Mcquitty clustering [44]. The results for models A, C and D (right) and models B, E and F (left) are shown in Figure 3. We used for the three major branches of the clusters three different colors to highlight them. The resulting clusters are not without error with respect to the types of the models. However, overall, the obtained clusters correspond well to models with different kinetics. Clustering all models together results in similar but slightly worse clusters.

The sensible cluster formations of our hierarchical clustering in Figure 3 demonstrate that time series data from different models carry indeed different information, which can be captured by our 10 features. This implies that our 10 dimensional feature vectors are sufficient to accomplish their separation. Further, it shows that the formation of these clusters is robust because the differences in the height of the lowest clusters compared to the major branches is up to 30-fold larger. This is confirmed by a Bootstrap analysis using only a subset of all available data to cluster the models, which leads essentially to the same cluster formations (not shown).

**Figure 4:**
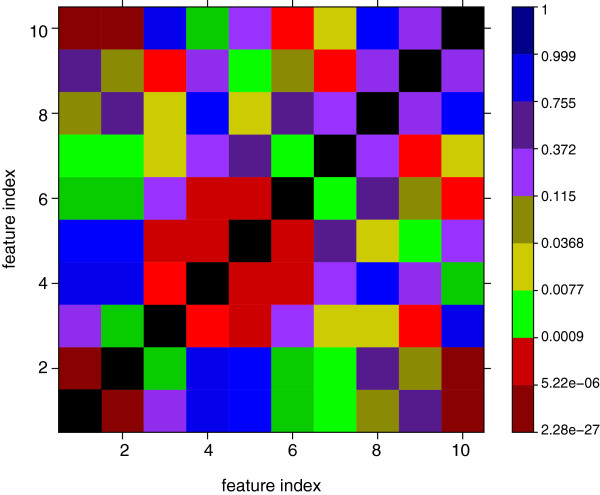
**Significance of correlations among features.** Graphical visualization of the p-values of correlation coefficients between different features. The colors red to blue represent low to high p-values. The diagonal is shown in black to indicate that the self-correlations are not of interest.

What the clustering in Figure 3 does not show is if all 10 features are actually required. For this reason, we repeated the clustering for many subsets of the 10 features and found always less meaningful clusters. This indicates that all features are different from each other and capture complementary information. To demonstrate this last point, we show in Figure 4 a graphical visualization of p-values obtained from correlation tests of the 10 features. More precisely, we use the same data we used for our clustering analysis and estimate the statistical significance of the correlation coefficients between the different features in form of p-values [39]. In Figure 4, the feature index corresponds to the feature number in Table 2. We use a color code for the obtained p-values where red indicates low and blue indicates high p-values (see the color key on the right side). Statistically, this means if a p-value is low (red) the correlation between two feature indices is high. Correspondingly, high p-values represent low correlation coefficients. Due to the symmetry of a correlation coefficient, the shown matrix of p-values is also symmetric. As one can visually see from Figure 4, the patterns demonstrate the independence of the features and explain why the removal of individual features worsens the clustering results. The mathematical interpretation of these results is that our feature vectors form a kind of base of the model space generated by the dynamical system we study.

Next, we quantify the classification abilities of the feature vectors. We use a random forest classifier (RFC) [45-47] to categorize all models. A RFC is an ensemble method that is based on decision trees. Due to the fact that it consists of multiple (usually thousands) decision trees and not just one, it is called a forest (of decision trees). Each decision tree is only capable of performing a linear classification, however, Breiman [45,46] showed that an ensemble of decision trees performs actually a non-linear classification. Training a RFC with 5,000 trees and averaging over 100 bootstrap [48] data sets results in a classification error of 11.75*%*(±0.37*%* standard deviation). If, instead, we are classifying models A, C and D and models B, E and F separately, then we obtain a classification error of 3.1*%*(±0.34*%*) for A, C and D, and 8.5*%*(±0.37*%*) for B, E and F.

Overall, these findings demonstrate that the information measured by the mRNA and protein numbers suffice to distinguish the models from each other, however, not without error. We studied many additional variables by enlarging the dimension of the feature vectors and found that the above classification errors can be further lowered. However, due to the moderate decrease in the classification errors (3*%*−4*%*) and the considerable increase in the complexity of the model (up to 24 dimensions), we focused on lower dimensional feature vectors as these suffice to provide affirmative information for our hypothesis.

### Estimating the location of a pause site

Finally, we estimate the location of a pause site from time series data. For this, we consider the *location of a pause site* as a *parameter* of the gene expression model and estimate its optimal value with a maximum likelihood estimation [49].

Because for the model of gene expression used here there is no known likelihood function available that could be used to obtain a maximum likelihood estimate for this parameter, we use an approximation thereof. The approximation proposed is based on the feature variables defined in Table 2, which have already proven useful for the clustering and classification of the models. Specifically, we define an *approximate likelihood function* as

(11) $$\begin{array}{lll} L(\theta |y) & =p(y|\theta )=\pi_{i=1}^{S}p(y_{i}|\theta )\; & \;\\ & =\pi_{i=1}^{S}\left(\right)\pi_{j=1}^{V}p(v_{j}(i)|\theta ))).\; \end{array}$$

Here, **y** is a *S*×*V*matrix where *S* is the sample size and *V * corresponds to the dimension of the random variable $y_{i}\in R^{V}$, which are the row vectors of matrix **y**. The components of **y**_*i*_, whereas the index refers to the *i*-th sample, correspond to the variables defined in Table 2, i.e., **y**_*i*_=(*v*_1_(*i*),…,*v*_*V*_(*i*)) with *V*=10.

For simplicity, we assume that the multivariate density *p*(**y**_*i*_|*θ*) can be written as the product of its components, i.e., $p(y_{i}|\theta )=\pi_{j=1}^{V}p(v_{j}(i)|\theta )$, implying the independence of *v*_*i*_from each other. In the previous section we saw that all random variables *v*_*j*_ are required to obtain a sensible classification of the models. This justifies the independence assumption, because if these variables were strongly dependent, the dimension of the feature vector could have been reduced.

Further, we define *p*(*x*|*θ*) as the joint probability density to observe the random variable *x*=*v*_*j*_ in the models $M_{\theta^{\prime }}$ and *M*_*θ*_. More precisely, the joint probability density is calculated by

(12) $$\begin{array}{lll} p(x|\theta ) & =\text{Prob}(x∼M_{\theta^{\prime }},x∼M_{\theta })=\; & \;\\ & =\int \min\{f(x|M_{\theta^{\prime }}),g(x|M_{\theta })\}\mathrm{dx.}\; \end{array}$$

Here, the probability densities *f * and *g* correspond to models $M_{\theta^{\prime }}$ and *M*_*θ*_, respectively. *f * and *g* are unknown and need to be estimated. We use a Gaussian density estimator [50] to estimate ${\hat{f}}_{N^{\prime }}(x|M_{\theta^{\prime }})$ and ${ĝ}_{N}(x|M_{\theta })$ from samples. The density ${\hat{f}}_{N^{\prime }}(x|M_{\theta^{\prime }})$ is estimated from the data **y**, with sample size *S*. In contrast, ${ĝ}_{N}(x|M_{\theta })$ is estimated from simulated data using model *M*_*θ*_ to generate data with sample size *S*^*′*^. Theoretically, *S*^*′*^≠*S* is possible, however here we used *S*^*′*^=*S*. The meaning of *p*(*x*|*θ*) is that, if *f*≡*g* then *p*(*x*|*θ*)=1. On the other hand, if $\min\{f(x|M_{\theta^{\prime }}),g(x|M_{\theta })\}=0$ for all *x* (*f * and *g* do not overlap) then *p*(*x*|*θ*)=0.

To motivate our approach, we note that the parameter *θ*^*′*^ in model $M_{\theta^{\prime }}$ corresponds to the *true* but unknown position of a pause site in the model from which we observed the data set **y**, and *θ* is the unknown position of a pause site in model *M*_*θ*_ that needs to be estimated. To estimate the probabilities, *p*(*x*|*θ*), in Equation 12, we simulate data from *M*_*θ*_for varying values of the parameter *θ*(position of a pause site). That means that we compensate for the lack of the availability of a likelihood function by the simulation of additional data sets to estimate some approximation thereof. When using only one variable, i.e., *V*=1, the likelihood function becomes *L*(*θ*|**y**)=*p*(*θ*|**y**) with $y\in R^{S}$. From this, the maximum likelihood parameter is estimated by $\hat{\theta }=\text{argmax}\{L(\theta |y)\}$. Because of the definition of the joint probability *p*(*θ*|**y**) (Equation 12), it follows that $\hat{\theta }=\theta^{\prime }$, which justifies its definition. For the multivariate case, the interpretation is similar.

**Figure 5:**
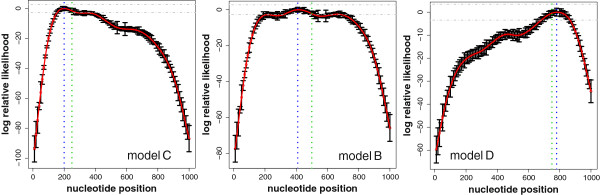
**Maximum likelihood estimation of the position of pause sites.** Logarithmic relative Likelihood for three models: Model C (left), model B (middle) and model D (right). The estimated maximum likelihood values of the nucleotide positions are 200, 410 and 780 (vertical blue lines) and the true position values (250, 500 and 750) are indicated by vertical green lines. The boundary of the 95% bootstrap confidence region of the ML estimates is indicated by horizontal lines.

Using this approach, we study if the location of the pause relative to the transcription start site (TSS) can be estimated from the time series measurements. In Figure 5 we show results of our analysis for models B, C and D. We show the *logarithmic relative likelihood* (LRL) [51] which is defined by

(13) $$\mathrm{LRL}(\theta )=\log(L(\theta ))-\log(L(\hat{\theta })).$$

The range of the LRL is from zero (maximum) to minus infinity. In the figures, the vertically dotted lines in green corresponds to the true but unknown position (*θ*^*′*^) of a pause site and the vertically dotted lines in blue are the maximum likelihood estimates of these positions. The error bars correspond to the standard deviation for the nucleotide positions estimated from *B*=50,000 bootstrap samples. All three maximum likelihood estimates ( $\hat{\theta }$) contain within their 95% bootstrap confidence region, shown as horizontally dotted lines, the *true* position of the pause site of models B, C and D.

Overall, due to the *likelihood principle*[52], our results justify the usage of Equation 11 as an approximate likelihood function.

## Conclusions

So far the identification of pause-prone sequences has relied on in vitro studies that make use of complex measurement procedures to characterize the kinetics of elongation of the RNAP [4,6,7,53]. These difficulties have hampered a proper assessment of possibly existing pause-prone sequences and thus a genome wide study of their role in the dynamics of gene regulatory networks. Further, there is a need for a better understanding of the role of these sequence-dependent and thus, evolvable events on the in vivo kinetics of gene expression.

Here we proposed a set of novel statistical methods that allow detecting the presence of pause sites, their location relative to the TSS, and their kinetics (mean duration), from time series data of mRNA and protein numbers at the single molecule level. This is motivated by the fact that such measurements are already possible to obtain in an almost genome-wide scale [14,18,20]. The methods proved to be efficient in all aims proposed when applied to a stochastic, sequence-level model of coupled transcription and translation in prokaryotes [15], found to be able to match measurements of gene expression at the single RNA and protein molecule level [18,20].

For the cases studied, there may be alternative features that perform better, in one sense or another. For example, to detect the existence of a pause site we used the mean RNA and protein numbers. This feature is only suitable if the induction level is strong enough for several collisions between RNAPs to occur during the simulations. Additionally, this feature is affected by the codon sequences, which here are randomly generated in each simulation. In this case, and for the realistic parameter values used, this feature proved to be sufficient. In other conditions, the use of different or additional features may be required.

At the moment there is no means to experimentally validate the results. For that, one needs to measure, in vivo, RNA numbers at the single molecule level. The MS2-GFP tagging system of RNA molecules is likely to not be usable, not only because it immortalizes the RNA, but it most likely affects the secondary and tertiary structure of RNA as the binding of MS2 is likely to hamper formation of structures such as hairpin loops, which are needed to confer transcriptional pauses with stability [4]. Instead, the best approach may be to engineer two genes that code for a tsr-Venus protein [20] and are under the control of the same promoter. In one of the genes, a his-pause would then be introduced, while the other would be used as a null model. Controlling the expression of these sequences, one should use a strong promoter, capable of transcribing RNA at a rate of 200 s^−1^ or faster (the lac promoter is a tentative choice [54]).

The methods used here require data from different models to compare them with each other. This is regardless of the type of the statistical method employed. For example, to detect whether a pause exists from real gene expression data, one must provide a certain amount of data of the dynamics of expression of a gene that indeed contains a pause and data of a gene that does not contain the pause. Similar data are required if one wants to determine the location of pause sites and their durations. Hence, regardless if a hypothesis test, clustering or a classification method is used, one needs data that can be *compared* with each other in a statistical manner. We believe that this is not a problem. It is feasible to engineer genes, with the same promoters as the native genes, while having elongation sequences that follow those requirements and are known. From the data resultant from these engineered genes, we can train the algorithms prior to providing data on the native genes that may or may not possess pauses. If these have similar kinetic properties to the pauses of the synthetic genes, their presence is bound to be identifiable by the trained algorithm.

From the above, the method proposed here to identify unknown pause-prone sequences is rather laborious on the experimental side. Nevertheless, it is feasible using known, relatively simple experimental techniques [14,18-20]. Also, to the best of our knowledge, it is the first method that can detect pause sequences from in vivo measurements of RNA and protein numbers. Finally, this method is not more extensive than the one presently used to detect pauses by in vitro techniques, which relies on the use of polystyrene beads held in optical traps [7].

A recent work [55] proposed a model of transcription elongation that allows, based on the DNA sequence, to predict to some extent the occurrence and duration of ubiquitous pauses. From measured rates of incorporation of nucleotides (that depend on the previously added one and on the one to be added), they derive a distribution of durations of these events for a certain DNA length. This distribution shows that some of these events can take several seconds to occur, thus providing an explanation for the occurrence of ubiquitous pauses during elongation. Unfortunately, the model is unable to predict long-duration pauses [55] as these are due to processes not accounted for by their model, such as the formation of hairpin loops in the elongating RNA and their interaction with the RNA polymerase, as in the case of his pauses [4]. Nevertheless, this approach, provided the inclusion of further details on the kinetics of transcription and translation (some of which may be unknown at the moment), may allow in the future to predict the occurrence of long pauses as well. In that case, the combined of use of this method along with ours (which allows determining the occurrence of pauses from the kinetics of RNA and protein production), may be of great aid in detecting and better understanding the nature of sequence dependent pauses.

In another work [56], a model of transcription elongation was proposed that was able to predict the kinetics of a specific type of transcriptional pauses, based on the sequence dependent translocation of the RNAp. Namely, the model accurately matched the kinetics of the tR2 pause [57]. It is yet unknown if this model can be extended to also be able to predict, from the DNA sequence, the occurrence of pauses such as the his pause, which require the formation of secondary RNA structures [4]. A similar work [58] proposed a method to predict sites for backtracking pauses. The method cannot be used for hairpin-induced pauses, since it cannot determine their stability. Again, in our understanding, these methods, provided their extension to include the long-duration pauses, can be used in parallel with the method proposed here since these methods aim to predict pauses from the sequences while we aim to detect the pause, its kinetics, and its location from RNA and protein numbers.

In conclusion, our methods provide means to detect unknown pause-prone sequences from temporal gene expression measurements and to determine their location in the sequence relative to the transcription start site and their kinetic properties. It may thus facilitate their identification from genome-wide temporal gene expression measurements. From this mapping, and by correlating these findings with the functions of the various proteins in the cells, we may enhance our understanding of whether and how this sequence-dependent mechanism is used in the regulation of genetic network dynamics [59,60]. Finally, this knowledge may aid in developing novel means by which one can regulate the degree of noise in the dynamics of engineered genetic circuits.

## Competing interests

The authors declare that they have no competing interests.

## Authors’ contributions

ASR and FES conceived the analysis. FES, AH and ASR performed the analysis and wrote the paper. All authors read and approved the final manuscript.
